# Lack of Detectable Allergenicity in Genetically Modified Maize Containing “Cry” Proteins as Compared to Native Maize Based on *In Silico* & *In Vitro* Analysis

**DOI:** 10.1371/journal.pone.0117340

**Published:** 2015-02-23

**Authors:** Chandni Mathur, Pooran C. Kathuria, Pushpa Dahiya, Anand B. Singh

**Affiliations:** 1 CSIR-Institute of Genomics & Integrative Biology, Delhi University Campus, Delhi, India; 2 National Allergy Centre, New Delhi, India; 3 Maharishi Dayanand University, Rohtak, Haryana, India; National Institute of Plant Genome Research, INDIA

## Abstract

**Background:**

Genetically modified, (GM) crops with potential allergens must be evaluated for safety and endogenous IgE binding pattern compared to native variety, prior to market release.

**Objective:**

To compare endogenous IgE binding proteins of three GM maize seeds containing Cry 1Ab,1Ac,1C transgenic proteins with non GM maize.

**Methods:**

An integrated approach of *in silico & in vitro* methods was employed. Cry proteins were tested for presence of allergen sequence by FASTA in allergen databases. Biochemical assays for maize extracts were performed. Specific IgE (sIgE) and Immunoblot using food sensitized patients sera (n = 39) to non GM and GM maize antigens was performed.

**Results:**

In *silico* approaches, confirmed for non sequence similarity of stated transgenic proteins in allergen databases. An insignificant (p> 0.05) variation in protein content between GM and non GM maize was observed. Simulated Gastric Fluid (SGF) revealed reduced number of stable protein fractions in GM then non GM maize which might be due to shift of constituent protein expression. Specific IgE values from patients showed insignificant difference in non GM and GM maize extracts. Five maize sensitized cases, recognized same 7 protein fractions of 88-28 kD as IgE bindng in both GM and non-GM maize, signifying absence of variation. Four of the reported IgE binding proteins were also found to be stable by SGF.

**Conclusion:**

Cry proteins did not indicate any significant similarity of >35% in allergen databases. Immunoassays also did not identify appreciable differences in endogenous IgE binding in GM and non GM maize.

## Introduction

Agricultural biotechnology has been used to engineer a variety of agronomically important crops, such as corn, soybean, potato, rice, maize, cotton and others. Genetically modified (GM) crops are produced by the insertion of specific genes that either encode a transgenic protein or express anti-sense RNA transcripts that inhibits viral infection or expression of any endogenous protein [1].GM crops that are resistant to insects have been produced by genetic modification with genes obtained from the bacterium *Bacillus thuringiensis* (Bt), and are reported to be commercialized since 1996 [2–3]. Biotech crops with Bt genes alone are reported to occupy 15% of the global biotech area, compared with 26% of stacked traits in major biotech maize producer countries as Brazil, USA, Argentina and Canada [4].

Bt has been shown to produce crystal proteins known as Cry proteins or inclusion bodies that are specifically effective in controlling certain orders and species of insect pests. Bt crystal proteins have been generally classified based on their insecticidal activity as Cry1, Cry2, Cry3 and Cry4. These proteins are reported to be toxic to lepidopteran, dipteran and coleopteran pests [2].

Safety and allergenicity of GM crops are required to be evaluated before market release as defined by scientific organisations such as International Life Sciences Institute/International Food Biotechnology Council, ILSI/IFBC [5], Food and Agriculture Organization/ World Health Organization, FAO/WHO 2001[6], Codex Alimentarius Commission 2003 [7], and Organization for Economic cooperation and Development, OECD 1998 [8]—to prevent the transfer of a gene encoding a major allergenic protein (from any source), into a food crop that did not previously contain that protein.

In India, the manufacture, import, use, research and release of genetically modified organisms (GMOs) as well as products are governed by the guidelines notified by Ministry of Environment and Forests (MoEF), RCGM-DBT (Review Committee on Genetic Manipulation, Deptt of Biotechnology), Govt. of India and Indian Council of Medical Research (ICMR, Food Safety guidelines 2008) [9].

An important step for novel protein safety assessment is to use bioinformatics tools. The two main uses of this approach is to assess whether the novel protein is a known allergen due to greater than 35% sequence similarity or is potentially cross-reactive with an existing allergen. However, *in vitro* tests are also suggested for analyzing any changes in the proteome make up of GM seeds due to post transgene integration and expression such as rice, potato and cotton [10].

Varieties of crops such as peanut, soybean along with its genetic variants have been highly focused for elucidation of allergenic nature since these are among the big eight foods known to cause food allergy [11]. Food allergy is estimated to be nearly 5% in adults and 8% in children, with growing evidence of an increase in their prevalence [12]. Clinical food allergy (based on Oral food challenge, OFC) in preschool children in developed countries is now as high as 10%. In large and rapidly emerging societies of Asia, such as China and Korea where there are documented increases in food allergy, the prevalence of OFC-proven food allergy is now around 7% in pre-school children, comparable to the reported prevalence in European regions [13].

If the modified plant is from a common allergenic source (e.g. soybean), recommended guidelines suggest testing for increase in the expression of endogenous allergens [14]. Some organizations in Japan and Europe have opted for evaluating endogenous allergens for rarely allergenic plants e.g. maize and rice [15].

Maize is not known to contain endogenous toxins or antinutritional factors till date as per literature search and reports on allergy to maize and its genetic variants in Indian context are still lacking.

The major objective of this study has been to assess the GM maize seeds containing Bt- Cry 1Ab, Cry 1Ac and Cry 1C protein sequences for allergenicity by *in silico* bioinformatic searches as well as by *in vitro* methods involving determination of specific IgE binding pattern to GM as well as non-GM maize antigens using maize sensitized patients sera.

## Materials & Methods

Three GM maize seeds containing Cry 1Ab, Cry 1Ac and Cry 1C transgenic proteins along with non-GM maize seed were procured from the developer, Metahelix Pvt. Ltd., Bangalore, India as per DBT guidelines.

The transgenes (events) incorporated in seeds were obtained from sources with no allergenic history and have been approved by regulatory agency RCGM-DBT, Govt. of India.

### *In silico* Analysis

**Selection of the query protein sequences**. Three insecticidal proteins of Bt—Cry 1Ab, Cry 1Ac, Cry 1C (Accession No—Uniprot P0A370,P05068, Q58FM0) which are preferable for development of genetically modified maize as mentioned in IGMORIS database were selected for present investigation.

**Sequence Database Selection**. All sequence homology search were performed against two allergen specific databases—Allergen Online of Food Allergy Research and Resource Program (FARRP) and Structural Database of Allergenic Proteins (SDAP) [16].

**Full Length FASTA Search**. FASTA comparisons are initiated by aligning the first match of a specific word size (*k-tuple* parameter, ktup = 2), followed by extending the alignment. Specific parameters used for this analysis included an expectation threshold of 10, a gap creation penalty of 12 and gap extension penalty of two. The BLOSUM matrix series was derived from a set of aligned, ungapped regions from protein families, called the BLOCKS database. The BLOSUM50 matrix identified blocks of conserved residues that are at least 50% identical. The extent of similarity was calculated as percent similarity and *E* score (expectation score). The *E* score reflected the degree of similarity between a pair of sequences based on matches of identical or functionally similar amino acids. A large *E* score indicated a low degree of similarity between the sequences under study and the sequence from the database and vice versa.

**80 amino acid Sliding window approach**. FASTA alignment was performed to compare all possible contiguous amino acid segments of each of the 3 proteins against the sequences listed in the databases. Each contiguous 80 amino acid sequence of individual protein was searched starting with 1–80 aa, then 2–81 aa, and so on until the last 80 amino acid segment of each protein is compared with database. In this search, % (percent) identities were calculated to evaluate possible cross allergenicity. An alignment of query sequence if showing >35% similarity over segments as short as 80 amino acids with known allergen(s) depicted that the query sequence might be a potential allergen and should be subjected to further *in vivo* and *in vitro* testing [10].

### Allergen Database

**AllergenOnline Database version 14.0**. The Food Allergy Research and Resource Program (FARRP) administered by University of Nebraska, Lincoln provides access to a peer reviewed allergen list, with 1076 protein sequences and sequence searchable database intended for the identification of protein that may present a potential risk of allergenic cross-reactivity. The objective was to identify proteins which might require additional tests, such as serum IgE binding, basophil histamine release or in vivo challenge to evaluate potential cross-reactivity. The database is updated annually and has added 66–85 known or putative allergen sequences (many of which are homologues of proteins al- ready included in the database) per year since 2007 [10]. The scoring matrix used on the AllergenOnline is BLOSUM 50 [17] that is weighted to favor identical matches between amino acids. Output of the FASTA was given as E-score.

**Structural Database of Allergenic Proteins (SDAP)**. SDAP is a Web server that links to the major protein (PDB, SWISS-PROT, PIR, GenBank) and literature (PubMed, MEDLINE) servers with 1526 Allergens and isoallergens. It uses an original algorithm based on conserved properties of amino acid side chains to identify regions of known allergens similar to user-supplied peptides or selected from the SDAP database of IgE epitopes. The major SDAP source of allergens is the IUIS list, http://www.allergen.org and alignments are made with FASTA v 3.45. The alignments between the query sequence and all SDAP allergens are reflected in terms of E score. Sequences with E values < 0.01 are almost always homologous.

### Bioassay

**Qualitative Evaluation of Transgenic proteins in GM maize seeds**. Qualitative GMO Check Bt test kit (KRISHILISA,Krishgen Biosystems, India) was used to evaluate the presence/absence of Cry 1Ab, Cry 1Ac as well as Cry 1C proteins in GM maize. The kit contained precoated microtitre plate containing antibodies to Bt-Cry1Ab, Cry 1Ac or Cry 1C protein along with positive control and extraction buffer. The kit employs the principle of sandwich ELISA of Antibody-Toxin-Enzyme labeled antibody. One twenty five milligrams of respective GM and non GM seed powder was resuspended in 1 ml of Extraction buffer (1X) of the kit provided. The seed suspension was mixed thoroughly and the solids was allowed to settle for 30 minutes and the supernatant was used for the assay. The manufacturer’s instructions were followed thereafter as described—50 μl of sample supernatant along with positive control and extraction buffer as blank were added to the precoated microtitre plate. The microtitre plate was mixed slightly and incubated for 15 min, at 37°C followed by addition of 50 μl of enzyme conjugate and incubation for 45 min at 37°C. The plate was then aspirated and washed 4 times with wash buffer (1X). Substrate solution 100 μl was added to all the wells in the plate and incubated for 15–30 min at ambient temperature in dark. Finally, stop solution (100 μl) was added to all the wells and absorbance was measured at 450 nm.

**Preparation of Maize Seed Extracts**. GM seed powders of maize containing Cry 1Ab, Cry 1Ac and Cry 1C protein along with the non-GM seed powder were suspended in 1:20 (w/v) ammonium bicarbonate buffer (50 mM, pH 8.0) with 5mM ethylene diamine tetra acetate and 1mM phenyl methyl sulfonyl fluoride, followed by continuous stirring for 6 h at 4°C as per standard protocol [18]. The extracts were centrifuged at 10,000g and the supernatant was filtered through a 0.45μm membrane filter. The filterate thus obtained, was lypohilized and stored in small aliquots at -70°C for further use.

The lyophilized extracts were reconstituted in 50% glycerinated phosphate buffered saline (PBS) (1:10 w/v) for use in SPT. A drop of the extract was placed on the volar aspect of the forearm and the skin was pricked by a 26 1/2″ G sterile needle. The SPT reactions were observed after 20 minutes and wheal diameter ≥ 3 mm were considered positive and graded from 1+ to 3+ based on wheal diameter [18].

**Protein Content and Profiling**. Protein content in the extracts of GM and non GM maize seeds were estimated by modified Lowrys method by precipitation of proteins using phosphotungstic acid [19].

Sodium dodecyl sulphate-polyacrylamide gel electrophoresis (SDS-PAGE) using 12% reducing gel was employed for obtaining protein profile of all the antigenic extracts followed by staining with Coomassie Brilliant Blue R-250, CBB [20].

**Simulated Gastric Fluid Digestion (SGF)**. Pepsin digestion assay was carried out to find out the stability of food protein under gastric fluid environment. The protocol of Astwood *et al*. [21] and Ofori-Anti AO *et al*. [22] with slight modification was followed. Briefly, a protein extract (680 μg) of each of the four maize seed extracts was treated with 200 μL of prewarmed SGF containing 0.32 w/v percentage of pepsin [23] in 0.03MNaCl, pH—1.2. Digestion was proceeded at 37°C with continuous shaking, and an aliquot (20 μL) of this digest was periodically withdrawn at 0, 1, 5, 15, 30, 45, and 60 minutes for analysis. These aliquots were quickly mixed with 26 μL of a sample buffer (containing 2% β-mercaptoethanol and 4% SDS) for SDS-PAGE together with 6 μL of Na_2_CO_3_ solution (200 mmol/L). As control, each protein sample was treated with SGF that did not contain pepsin and processed as described above. Breakdown of GM and non GM seed proteins was evaluated by SDS-PAGE using 12% acrylamide gel.

### ImmunoAssay

**Ethics Statement**. The study protocol was approved by Institutional Human Ethics Committee. Informed written consent was obtained from patients and controls subjects for participating in the study.

**Study Subjects**. A total of 39 patients of suspected food allergy by history and Skin Prick Test (SPT) from two allergy clinics located in North India (PC) were selected for the immunoassay. The mean age of patients was recorded as 35 ± 2.3 yrs. All the patients recruited in the study showed SPT positivity to one or the other common food allergens including five foods selected for detailed study (Table A in S1 File). Blood samples were drawn from all food sensitised patients (n = 39) as well as healthy volunteers (n = 11) who served as control.

**Estimation of Specific IgE (sIgE) to Maize antigens**. Serum IgE against three transgenic maize as well non-transgenic maize extracts, was estimated in the sera of all 39 patients as well as 11 healthy volunteers by Enzyme linked Immunosorbent Assay (ELISA) following the protocol of Sepulveda *et al*. [24]. Protein extract(s) in carbonate buffer (0.2 μg/100 μl per well) was coated in a microtitre plate (Nunc, USA) and kept overnight at 4°C. After repeated washing with phosphate buffer, the nonspecific sites were blocked with 1% Non-fat dry milk for 2 hr. at 37°C. The plate was washed and incubated with 1:10 v/v diluted sera of patients, overnight at 4°C. The plate was washed with phosphate buffer saline-Tween 20 (PBST) usually four times (each wash 3 min) and incubated with monoclonal antihuman IgE-horse radish peroxidase (1:1000 v/v; Southern Biotech, USA) in 0.05% non-fat dry milk in PBST for 4 h at 37°C. Colour was developed with orthophenylene diamine after 4–6 min. The reaction was stopped by adding 50 μl 2.5 M H_2_SO_4_ and the absorbance was read at 492 nm and the value of different serum samples was analysed according to the criteria of Kauffman *et al* [25].

**Immunoblot**. The SDS resolved proteins of non GM and GM maize antigenic extracts on 12% acrylamide gels were transferred on to nitrocellulose membrane (NCM) as described by Towbin *et al* [26]. The unbound sites were blocked by 3% Non fat dry milk for 3 hr at 37°C. The NCM strips were washed and incubated with 1:10 v/v food allergic sera at 4°C overnight. Healthy serum pool was taken as control. The strips were washed with PBST and incubated with 1:1000 diluted antihuman IgE-peroxidase. The IgE binding was detected by diaminobenzidine (DAB) with hydrogen peroxide in sodium acetate buffer (pH 5.0). The detection was finally stopped using 0.2N HCl.

### Statistical Analysis

All statistical analysis were performed using MS-Excel and Prism V software (Graph Pad Prism, San Diego, California, USA). Values are represented as Mean±SEM(standard error mean). The significance level was considered to be p<0.05 (two-tailed).

Qualitative Interpretation for confirmation of Cry proteins in transgenic/GM seed powder was based on absorbance/Optical Density greater than the cutoff value (Mean for Blank + 0.1) obtained in ELISA.

ELISA based specific IgE values of sera samples were analysed as per Criteria of Kauffman *et al*. [25] with slight modifications. Serum IgE percent binding for each of the 39 sample relative to control sera(pool) and positivity were calculated as follows:

$$\text{Percent Binding}(\%)=\frac{\text{OD of individual patient sera—OD of control sera}}{\text{OD of highly positive sera—OD of control sera}}\text{×100}$$

OD values depending on per cent binding were categorized into 4 groups i.e < 15, 15–30, 30–60 and > 60%. Sera showing more than 30% binding were considered to have significantly raised (p < 0.05) specific IgE against respective non GM and GM maize antigens.

## Results

### *In silico* Analysis

A comparative bioinformatic approach of sequence homology have been used, for assessment of allergenicity of Cry proteins. The length of three transgenic Cry protein sequences is—Cry 1Ab 1155 amino acid(aa), Cry 1Ac 1178 aa while Cry 1C 1044 aa. The sequences of the proteins have been tested against the AllergenOnline and SDAP databases. The result of bioinformatic searches are compiled and analysed as provided in Table 1.

**Table 1:** Sequence homology of selected sequences using FASTA under different allergen databases.

Query Sequence	Length (aa)	Accession No.	Full Length FASTA Search	Full Length FASTA Search	80 amino acid sliding window approach	80 amino acid sliding window approach
			Allergen Online	SDAP	Allergen Online	SDAP
Cry 1Ab	1155	P0A370	No sequence with E score < 1.0	6%,Asp f 5 (CAA83015)	No hits > 35% identity found	32%,Mala s1 (Q01940)
				4%,Mala s1 (Q01940)		27%,Glym1(AAB09252)
				3.6%,Tri a gliadin (AAA34285)		26%,Lig v1 (O82015)
				2%,Pench 20.0 (AAB34785)		24%,Bosd 8 (AAA30478)
Cry1Ac	1178	P05068	No sequence with E score < 1.0	3.7%,Tri a gliadin (AAA34285)	No hits > 35% identity found	30%,Asp f 13 (P28296)
				3.6%,Ana c 2 (BAA21849)		25%,Lig v1 (O82015)
				6%,Chit 6.0(P02223)		23%,Tri a gliadin (AAA34285
				2.7%,Phl p 5.0(CAD87529)		24%,Ani s 2 (AAF72796)
Cry 1C	1044	Q58FM0	E score = 0.9 with 29 kD IgE binding protein from *Candida albicans*	4.7%,Hev b 9 (Q9LEJ0)	No hits > 35% identity found	33%,Cor a1.0 (CAA96549)
				4%,Tri a 12 (P49232)		

**The Bioinformatics Assessment of Cry proteins using FASTA 35 against the FARRP AllergenOnline**. The full length FASTA alignment as well as sliding 80 mer window approach for the protein sequence of Cry 1Ab, Cry 1Ac as well as Cry 1C in AllergenOnline database was assessed. Both the tests were performed using the default settings of E value (< 1) and Z score for full length FASTA and a similarity of ≥ 35% over 80 amino acid stretch (Table 1). The results obtained on assessment for the proteins revealed that transgenic protein Cry1Ab and 1Ac did not reflected any sequence similarity with any known allergens due to E score >1. On the other hand, the Cry 1C protein exhibited an E score = 0.9, and only 26% full length identity with a fungal protein of *Candida albicans* (as in Table 1), which is well below the threshold of greater than 50% to be assumed a allergenic sequence as suggested by Aalbrese (2000) [27].

Sliding 80 mer window approach of the FARRP didn’t presented any segment in any of the three Cry protein sequences with over 35% similarity to any known allergen. This results also did not adhere to the criteria as laid down by Codex for positivity of allergenic cross reactivity by any of three transgenic proteins (Table 1).

**Bioinformatic assessment of the Cry proteins in Structural Database of Allergenic Proteins (SDAP)**. Protein sequences of Cry 1Ab, Cry 1Ac and Cry 1C were inspected for presence of allergenic segment in another allergen specific database—SDAP. Full FASTA (v 3.45) alignment for the Cry protein sequences was analysed for sequence homology against all the allergens in SDAP with the default parameter to show sequences with E score ≤ 0.01. FASTA alignments using sliding 80 mer window for identifying ≥35% similarity region of amino acids was also observed for three Cry proteins under study in SDAP.

The maximum similarity in full length alignment for the three Cry proteins was obtained as percentage similarity in the range of 6–4%, as described below and depicted in Table 1.

**Cry1Ab**. Full FASTA alignment of the protein yielded sequence similarity of the range 6–2% with allergens of fungal origin such as Asp f 5 (*Aspergillus fumigates)* and Pench 20 (*Penicillium chrysogenum)* as well as food allergen Tria gliadin from *Triticum aestivum*. Under the 80 amino acid sliding window, a highest of 32% identity with Mala s 1 a reported allergen from *Malassezia synpodialis* was observed, followed by 27% sequence homology with Glycine max. allergen *Gly m* 1 *and* 24% with Bos d 8 from *Bos Taurus*. Plant derived allergens were more frequent in the compiled matching sequences.

**Cry 1Ac**. The highest scoring identity for the protein under Full FASTA alignment was found to be ~3–2% with different proteins—Tri a gliadin, Anac 2 (Bromelain) from *Ananas comosus*, globin named Chit 6 from *Chironmus thummi*, Ves v 2.0, an insect origin Hyaluronidase and timothy grass allergen named Phl p 5. The same protein sequence (Cry 1Ac) under 80 amino acid window showed a highest of thirty percent identity with Asp f 13, a fungal alkaline proteinase from *Aspergillus fumigatus* followed by 25% identity with Lig v1(pollen allergen) and 23% with Tria gliadin (food allergen).

**Cry 1C**. The best scoring identity observed under Full FASTA alignment was found to be 4.7% with reported allergen Hev b 9 from *Hevea brasiliensis*, 4% with fungal peroxisomal protein, Cand a 3 as well as food allergen Tri a 12 from wheat and ~ 3% with Pasn 1,a grass allergen and Bos d 8 (animal allergen). Under the 80 aa window approach highest identity of 33.7% with Cor a1, pollen allergen from *Corylus avellana* was recorded, 27% with Cand a 3 and 26% with Chaf 1, crab tropomyosin as well as Pasn 1, a plant protein was observed.

Specifically comparison of Cry 1C analysis for sequence homology in FARRP and SDAP database presented similarity to a common known allergen from *Candida albicans*. But the E score was almost equal to the threshold in Full FASTA alignment and percentage similarity was below than required, the probability of assuming Cry 1C protein as allergenic was nullified. Several sequences with decreasing similarities in all the above cases were observed which are not described here.

**Fig 1:**
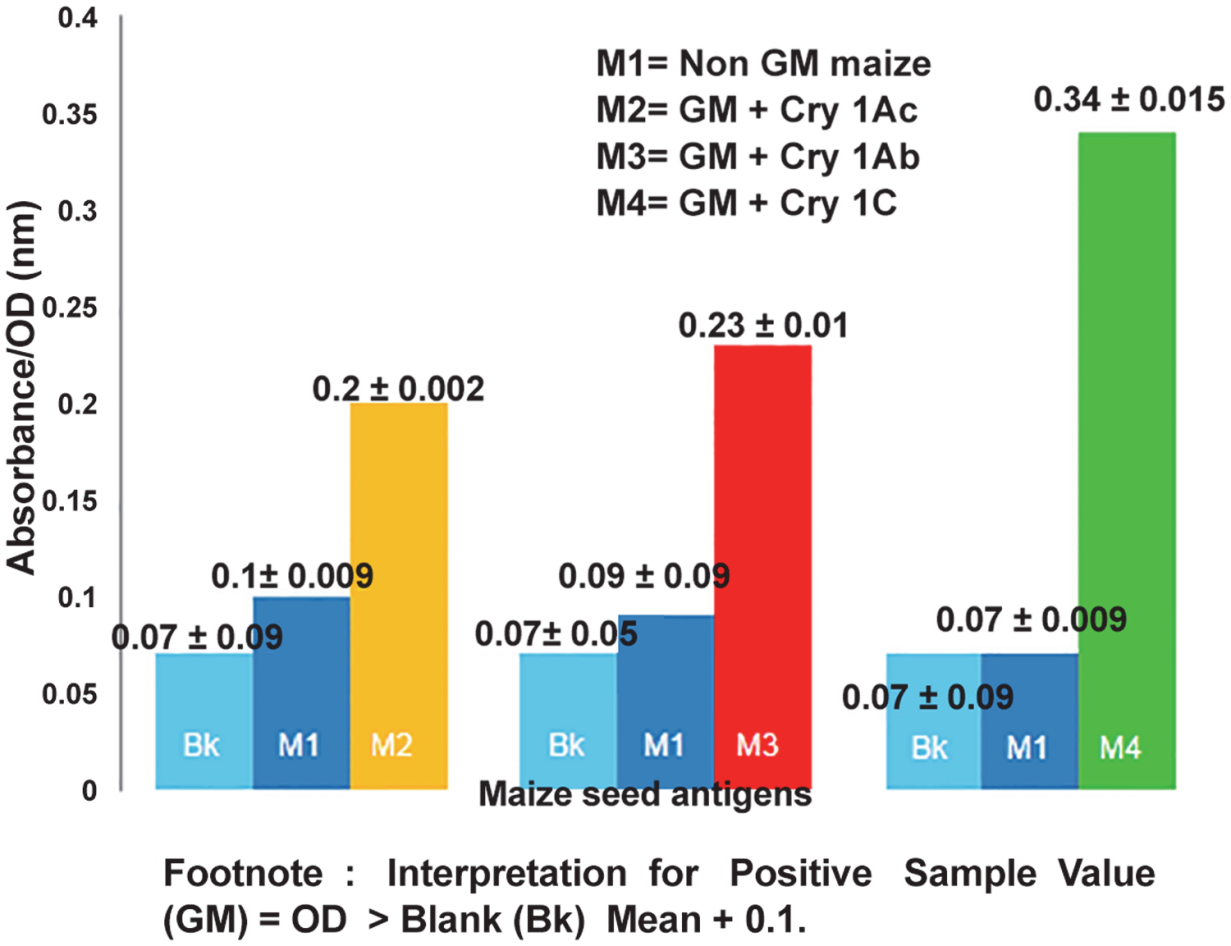
Qualitative ELISA based Confirmation for Presence of Bt-Cry proteins in GM maize seed powders. Absorbance/OD values are presented as Mean ± SEM. Interpretation for Positive Sample Value (GM) = OD > Blank (Bk) Mean + 0.1.

**Quality Analysis of Cry proteins in procured maize seeds**. Presence of Transgenic proteins Cry 1Ab, Cry 1Ac and Cry 1C in respective GM maize powder extracts were detected by Qualitative ELISA Kit as stated in methodology. Extraction buffer served as blank while Non GM maize seed, serving as negative control was also assessed for absence of any transgenic protein, by the same protocol. The higher the values for absorbance in ELISA for GM maize, above the described cutoff under section material & methods, the greater the concentration of respective transgenic protein. The absorbance values for GM and blank in each of three cases recorded were as follows- GM with Cry 1Ac and blank were 0.2 ± 0.002, 0.07 ± 0.09; GM with Cry 1Ab and blank were 0.23 ± 0.01, 0.07± 0.05; GM with Cry 1C and blank were 0.34± 0.01, 0.07± 0.09. Since, the absorbance values for GM sample in each of the three cases recorded were greater than the desired criteria, this leads to confirmation for presence of respective transgenic proteins, Fig. 1.

**Protein Assay**. Protein content in three GM maize antigens was estimated as—Cry 1Ac with 18 mg/gm dry wt., Cry 1Ab with 19 mg/gm dry wt., Cry 1C with 15 mg/gm dry wt. and and in non GM maize as 20 mg/gm dry wt. Thus, there was no significant variation in protein content of three GM maize extracts and non GM extract (p> 0.05).

**Fig 2:**
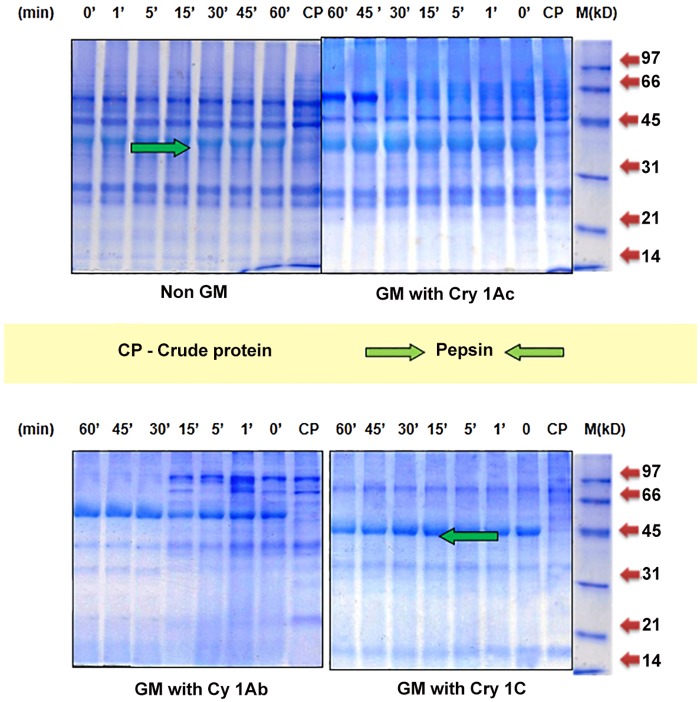
Analysis of stable protein fractions profile in different maize seed extracts by SGF over 60 min of pepsin digestion on 12% SDS-PAGE.

**Simulated Gastric Fluid Digestion Assay**. The digestibility of proteins in non GM and GM maize antigenic extracts revealed changes in the protein profile after pepsin treatment on 12% SDS-PAGE. Pepsin digested stable protein fractions are defined as reluctant to breakdown to short protein fragments with persistence as intense bands on gel. (Fig. 2)

In the non transgenic maize, out of the 12 protein fractions present in undigested sample- 6 protein fractions of 60, 38, 28, 19, 14 & 10 kD were observed as stable even after 1 hr of digestion while remaining high molecular weight six fractions- 152, 120, 105, 90, 80, & 40 kD were partially digested with very faint appearances in gel after 1 hr. In GM maize with Cry 1Ac, out of 12 protein fractions as observed in non-treated extract, 5 fractions—66, 58, 34, 15 & 9 kD were observed as stable even after 1 hr of pepsin digestion while rest 7 protein fractions- 175, 154, 126, 98, 80, 22 & 6 kD were found to be digested within 30 min of initiation of reaction. Further, in GM maize with Cry 1Ab, out of the total 13 protein fractions of the range- 170–7 kD, five fractions—68, 36, 30, 25, 16 were observed as stable while remaining8 fractions-170, 154, 133,107, 97, 82, 51 & 7.7 kD were digested completely within 1 hr.

In the GM with Cry 1C, 12 protein fractions were observed in undigested extract. Of these, five fractions of 67, 31, 28, 17 & 14 kD were found to be stable to pepsin digestion even after 60 min of digestion while remaining seven protein fractions- 160, 134, 118, 90, 77, 59 & 8.5 kD were completely digested within 15–30 min. of initiation of digestion.

**Fig 3:**
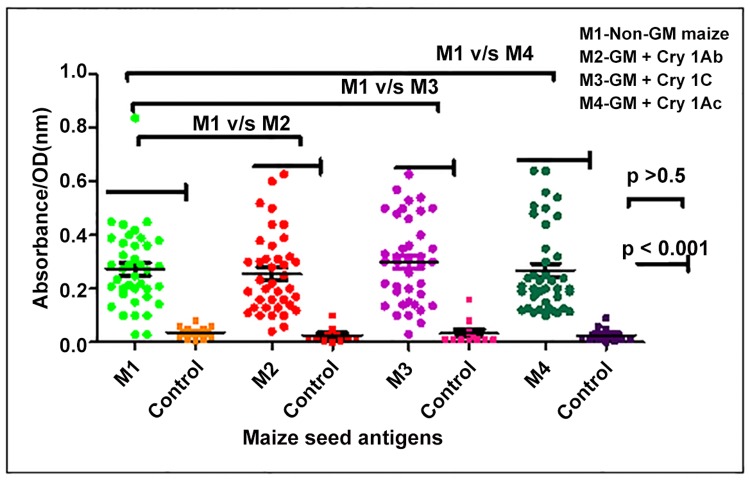
Specific IgE titres (OD values) against GM and non GM maize antigens in the sera of n = 39 respiratory allergy patients showing suspected food allergy. Sera from 11 healthy volunteers (control) were also assessed for specific IgE against all maize antigens. A statistical significant difference (p< 0.05) in specifc IgE values of patients v/s control was observed against the non GM and GM maize antigens. A statistical similarity (p> 0.5) of specific IgE values of patient group against the non GM v/s GM maize antigens (i.e intraspecies comparison) was observed.

**Maize Specific IgE (sIgE) in Patients Sera**. In the sera of 39 suspected food allergic patients and 11 control subjects, specific IgE against non-GM as well as three GM maize extracts was analyzed by ELISA in terms of absorbance/ optical density (OD) and further categorized according to percent binding criteria of Kauffman *et al* [25] (Fig. 3, Table B in S1 File).

The mean OD values of specific IgE of patients & control subjects against non GM maize were 0.27± 0.02 & 0.03±0.007, in GM maize with Cry 1Ab as 0.25± 0.02 & 0.02 ± 0.008, 0.3 ± 0.02 & 0.033 ± 0.014 against GM maize with Cry 1Ac and in GM maize containing Cry 1C as 0.26 ± 0.02 & 0.02 ± 0.008. A statistically significant difference of specific IgE values was observed in patients v/s control (p < 0.001) with respect to described four groups. Thereby signifying raised IgE levels in patient group against non GM and GM maize antigens under study (Fig. 3).

Further, on the basis of specific IgE values against non GM and GM maize antigens of patient group only, variation among maize antigens (intra species) viz non GM v/s GM maize with Cry 1Ab, non GM v/s GM with Cry 1Ac, non GM v/s GM with Cry 1C was observed to be statistically non significant (p > 0.5). Thus, similarity of specific IgE values of the patients was observed against the non GM as well as GM maize antigens.

In Non GM maize, OD values of the patients ranged from 0.03–0.83 while in the control subjects it ranged from 0.004–0.08. Among the 39 patient sera, 16 showed IgE binding of < 15% as compared to control and were considered showing low specific IgE. Non- GM maize specific IgE of 15–30% was observed in 15 cases and were considered moderately positive. However, serum samples from 9 cases showed more than 30–60% specific IgE binding and selected for further immunoassay studies.

**GM maize with Cry 1Ab**. All the 39 patients reflected OD values of specific IgE in the range of 0.04–0.6 while in the control subjects the range was 0.0–0.10. Out of the total 39 cases evaluated, 19 showed IgE binding of < 15% as compared to control and were considered showing low specific IgE. GM containing Cry 1Ab maize specific IgE of 15–30% was observed in 11 cases and were considered moderately positive. However, serum samples from 9 cases showed 30–60% specific IgE binding.

**GM maize with Cry 1Ac**. The OD values for specific IgE for 39 patients ranged from 0.03–0.60 while in the control group, the range was 0.008–0.16. Amongst the 39 patients, 16 showed IgE binding of < 15% as compared to control and were considered showing low specific IgE. GM maize specific IgE of 15–30% was observed in 12 cases and were considered moderately positive. However, serum samples from 11 cases showed 30–60% specific IgE binding.

**GM with Cry 1C**. All the 39 cases reflected OD values of specific IgE in the range of 0.10–0.64 while in the control subjects the range was 0.001–0.09. Out of the sera of 39 cases evaluated, 21 showed IgE binding of < 15% as compared to control and were considered showing low specific IgE. GM maize containing Cry 1C specific IgE of 15–30% was observed in 9 cases and were considered to be moderately positive. However, serum samples from 9 cases reflected 30–60% specific IgE binding while no single sera sample showed greater than 60% binding and a total of 9 sera were selected for further immunoassay.

Thus, out of the 39 sera samples analysed for specific IgE, the same sera of 9 cases reflecting significantly high specific IgE binding of 30–60% against non GM and GM maize antigens were selected. Out of these nine, selective five maize sensitized cases with SPT to maize as well as suspected food allergy were considered for immunoblot. The five patients were all adults varying from 18–53 yrs.(mean age 33 ± 6.3) of age with 1:4 male/female ratio. They suffered either from nasobronchial allergy, urticaria, diarrohea or oral allergy syndrome etc. These patients had recurrent symptoms whenever they consumed corn “roti” or roasted maize preparations. Their symptomatic treatment included antihistamines, bronchodilators and corticosteroids etc. (Table 2).

**Table 2:** Clinical details of five patients of maize allergy with SPT positivity to maize elected for performing Immunoblot.

Patient No.	Age/Sex	Symptoms appeared after(minutes) food ingestion	No. of episodes (per year)	Symptoms of maize allergy*	Prescribed Medication
1.	42/F	30–40	4–5/year for 8 years	Nasobronchial Allergy	Formoterol, Budenoside, Ciclesonide, Nasal sprays, Montelukast
				headache, nasal congestion, sore throat	
2.	18/M	15–30	2–3/year for 2 years	Urticaria, Angioedema	Anthihistamines
3.	31/M	15	2–3/for 1year	Nasobronchial allergy, Case of polysensitization	Montelukast, bronchodilators
4.	23/M	30–60	2–3/year for 1 year	Oral allergy syndrome, known case of suspected hypersensitivity to various foods as spicy and Chinese foods, icecream, vinegar, corn, tomato, cold-drink, throat infection, headache, swelling behind ears	Bronchodilators
5.	53/M	30–60	6–8/year for 15 years	Allergic rhinitis, Oral allergy syndrome	Bronchodilators with inhaled corticosteroids

* Also tested for other suspected foods.

**IgE Immunoblot Reactivity Assay**. The identification of IgE binding proteins in non GM and GM maize antigenic extracts was carried out by Immunoblot. Out of the 39 suspected food allergy/sensitized patients sera analysed for specific IgE so far, sera from five shortlisted maize sensitized cases (specific IgE positive) along with pooled sera of control subjects were further used to perform Immunoblot against non GM maize as well as GM maize antigenic extracts.

On case to case basis, heterogeneity of sera samples in binding to different protein fraction(s) of non GM and GM maize extracts was reflected. Protein fraction at 28, 33, 78 kD were unique to Case No. 1, 4 & 3 respectively while protein fractions at 41& 48kD were recognized in both Case No. 2 & 5, accounting in 40% of cases. Case No. 5 also showed binding of 68 & 88 kD protein fractions also.

**Fig 4:**
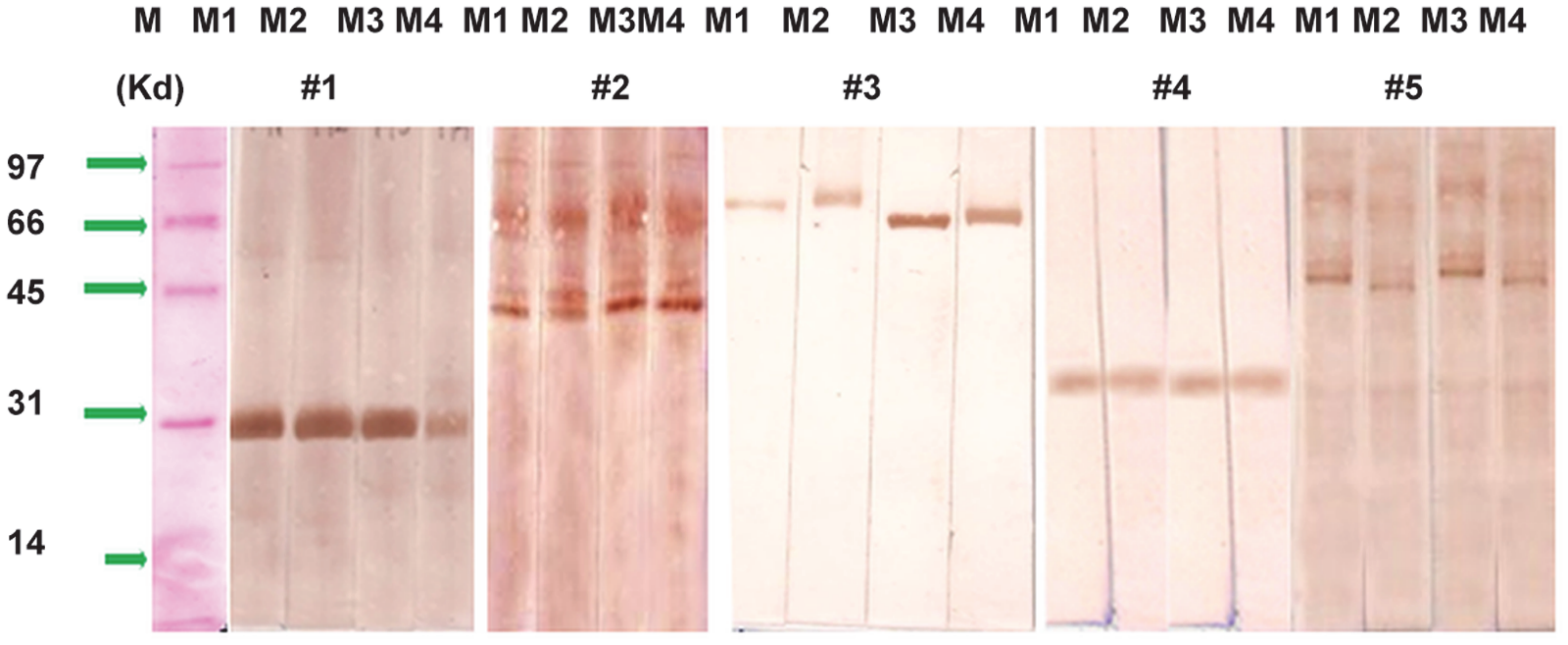
Immunoblot against against non GM and GM maize antigens in the sera of selected 5 maize sensitized patients using sera conc. of 1:10 v/v, to reveal IgE binding protein fractions. M1- Non GM maize, M2- GM + Cry 1Ac, GM + Cry 1Ab, M4—GM + Cry 1C. M- Protein marker.

**Comparative analysis of IgE binding among non GM and GM maize**. In non GM maize extract, 7 protein fractions of 28 (smear), 33, 41, 48,68, 78 and 88 kD approx. were recognized as IgE binding by selected five sera samples on Immunoblot. These same seven fractions were also observed as IgE binding by the same five cases in GM maize extracts of Cry 1Ab, Cry 1AC and Cry 1C respectively. This signifies absence of variation in IgE binding by selected sera samples against non GM and GM maize antigen extracts (Fig. 4).

## Discussion

Food is one of the basic requirement without which survival is at stake. In India, food production capacity has to be substantially raised to meet growing demand for human food and a falling ratio of arable land population. The major concern for farmers is to protect the crops both during pre and post harvesting conditions. To address these major concerns, varieties of crops are successfully engineered through genetic engineering i.e GM crops to improve crop quality, enhanced pest resistance, reduced dependence on pesticides, increased nutrient component, and production of pharmaceuticals etc. The same desirable traits may also be achieved through conventional breeding but it has limitations in terms of long duration for development, species barrier, concern for pesticide residue in crops and as a result genetic engineering has taken advantage for development of GM crops. This “gene revolution” as compared to “green revolution” is poised to benefit both poor and rich farmers equally and has an immense potential in transforming global and local agriculture.

Cereals are the major energy constituents of Indian food diet with maize being the third most important cereal after wheat and rice. In the past, efforts to increase maize production have been productive but there have been reports on annual loss of maize [28] of about 13.2% due to diseases. In India, spotted stem borer (*Chilo partellus*) and *Sesamia inferens* are serious pests causing diseases as stalk and ear rots of maize. It is very difficult to control this pest using contact insecticides since it bores into the stem and feeds on tissue affecting the growth of plant [29].

The establishment of the All India Coordinated Maize Improvement Project (AICMIP) in 1957, had led to significant progress in maize breeding with development of a large number of hybrids and composites with high grain yield, appropriate seed maturity, pesticide resistance etc.

However,the progress in breeding for insect resistance has been slow due to lack of effective insect pest rearing methods and germplasm screening technique [29]. This issue is being addressed and resolved through the approach of biotechnology, leading to development of GM maize containing traits such as insect resistance due to expression of Bt cry genes (Cry proteins) and herbicide tolerance through enzyme 5-enolpyruvylshikimate 3-phosphate (EPSP) synthase transgene by Indian seed developers. Cry proteins in GM crops are expressed as large insoluble proteins aggregates, in normal conditions and are safe for humans, higher animals and most insects but become solubilized as active toxin in reducing conditions of high pH = 9.5, as found in mid gut of lepidopteran larvae feeding on Bt Crops which leads to killing of these pests. These characteristics favour long term environmental friendly use as transgenic proteins [30].

Batista *et al*. [31] studied the allergenicity of three GM maize varieties expressing different levels of Cry 1Ab protein, based on SPT and Immunoblot reactivity assay in two groups of sensitive population with the assumption that participating population were consuming maize derived products from a GM maize source. Their study was carried in 2004 while the selected GM materials were commercialized by European Union since 1998. The same group concluded for safe nature of Cry 1Ab because of absence of detectable differences in IgE reactivity between GM and non maize. Buzoianu *et al*. [32] and Walsh *et al*. [33], have reported for non significant effect on growth and immune response of animals feed with GM maize (Bt-MON810) expressing Cry 1Ab and ruled out for any possibility of *cry1Ab* gene or protein translocation to the organs and blood at any stage of life cycle.

As per the guidelines of national and international scientific agencies such as ICMR-DBT, Codex, GM crops (before commercialization) need to go through a safety and allergenicity assessment for considering it is as safe as its traditional counterpart. In India, Bt cotton is the only commercialized crop till date. As per the data of IGMORIS [29], many GM maize crops with Cry proteins are under field trials and need to be evaluated for safety aspects.

To fill the lacunae of limited information on allergenicity to maize, the present work is targeted at comparing the IgE binding profile of GM maize containing Cry proteins with the non-GM maize using selected maize sensitized sera of Indian patients. The objective of the study has been achieved with a combination of *in silico* and *in vitro* tools. Cry protein sequences have been assessed for sequence similarity to known allergens using FASTA based bioinformatics tools for both full length sequence similarity as well as using short stretch of 80 amino acid window with cutoff criteria of > 35% sequence similarity [2, 5, 27] and found that these proteins are of non-allergenic source. Our findings are in accordance with an earlier study by Verma *et al*. [34] reporting for validation of the safety aspect of Cry 1Ab and Cry 2Ab inserted proteins sequences using *in silico* tools and predicted them as safe for genetic incorporation in plant kingdom. Similarly, Randhawa *et al*. [35], also reported that in various Cry group of proteins viz Cry1Ac, Cry1Ab, Cry2Ab, Cry1Ca, Cry1fa/Cry1Ca which are being incorporated in Bt Crops indicated no significant alignment and similarity at domain level with allergens, thus revealing no potential risk of allergenic cross-reactivity. Thus, our findings by *in silico* analysis on three Cry proteins confirm the findings of earlier workers [34–35] for incorporation in GM maize.

The second aspect of our study has focused on *in vitro* analysis to compare for changes in endogenous protein expression level due to incorporation of Cry 1Ab, Cry 1Ac and Cry 1C transgenes in maize seeds.

Melinda *et al*. [36] reported on Glyphosate-Tolerant Soybeans and its parental variety for determination of basic nutritional and biologically active components over three years of field testing. Analytes such as fat, protein, carbohydrates, isoflavones were determined and it was reported that the genetically engineered soybean with glyphosate tolerance was substantially equivalent to the conventional soybean. Similarly Noteborn *et al*. [37] studied safety and toxicity assessment of transgenic tomatoes expressing Bt Cry 1Ab protein, and observed no major changes in nutrient composition by chemical analysis between the transgenic and the native variety.

The variability in protein content among the three GM maize varieties and the non GM variety evaluated by us did not show any statistically significant variation (p> 0.05). Thus, absence of significant variability in protein content among GM and parental variety of maize are in synchrony with earlier reported studies [36–37].

Codex and the European Food Safety Authority have stressed on the potential that a newly expressed protein might become an allergen should be evaluated based on resistance to digestion in pepsin and abundance in food fractions [14]. However, each country has modified the testing criteria as per their regulations and policies but still adhere to Codex guidelines for food safety to facilitate international trade.

The simulated gastric model also provides a method to assess allergenic potential of endogenous proteins upon introduction and expression of foreign gene (with no allergenic history) such as Bt Cry genes, CP4 EPSPS, into food plants. Purified proteins and stained gel have been emphasized for analysis for stability in SGF by Astwood *et al*. [21]. Purified commercial preparations of various potential transgenes have been analyzed for IgE binding using sensitized human sera samples also. Batista *et al*. [31] have performed for SPT and IgE immunoblot reactivity assay on pure Cry 1Ab and CP4EPSPS proteins using selected maize and soybean sensitized patients sera and observed for absence of binding to these control proteins by any of the sera.

The relative stability profile on SDS-PAGE of pepsin digested antigenic extracts of GM and non GM maize seeds revealed different stable fractions. Higher molecular weight protein fractions mainly above 70 kD in all the three GM maize antigens were found to be more susceptible, as lesser time duration was required for digestion as compared to proteins above > 70 kD present in non GM maize which were observed to be partially digested as evident from faint appearance even after 1 hr. of digestion (Fig. 2). Protein fractions which were reluctant to digestion and interpreted as “stable” in the non GM and GM maize extracts were observed to be in the range of 68–9 kD. Six stable protein fractions of the range 60, 38, 28, 19, 14 & 10 kD were observed to be stable in non GM maize antigen. In the GM maize with Cry 1Ac, 5 protein fractions of the range 66–9 kD were observed as stable fractions while in the GM maize with Cry 1Ab five stable protein fractions—68, 36, 30, 25 & 16 kD were observed. Also, in GM with Cry 1C, five stable protein fraction of the range 67, 31, 28, 17, 14 kD were recorded. Thus, there seems to be no overall difference in protein profile of the three pepsin digested GM maize antigens. Presence of a extra protein fraction in addition to five stable protein fractions in non GM maize antigens as against five in GM maize antigens suggest that incorporation of transgene in maize genome might have resulted in change of the constituent expression of protein fraction of the range 68–9 kD which has led to alteration (reduction) in number of protein fractions within similar molecular weight range.

No identifable differences in allergic potency by specific IgE and Immunoblot as well as changes in endogenous proteins of genetically engineered soybean varieties containing glyphosate tolerant genes as against wild varieties have been reported by Burks and Fuchs [38]. In a study by Satoh *et al*. [15] on salt soluble protein extracts of non-transgenic and transgenic rice, patients revealed few qualitative differences between the IgE-binding proteins of the two extracts by 1D immunoblot. The antigen-specific-animal sera show no qualitative or quantitative differences in two known allergens of rice, RAG2 and glyoxalase I as well as novel proteins at 19, 52, and 63 kD. Metcalfe *et al*.[5] recognized that for less commonly allergic sources such as rice or corn, five test sera may be sufficient for an adequate safety test. In the present investigation, the variation in the specific IgE values against the transgenic and non transgenic maize antigens from the sera of 39 food sensitized patients were statistically non significant (p >0.05). This signifies that three GM maize antigens under analysis does not differ in the ability to bind serum IgE as presented by non GM maize, similar to earlier reported observations [15,31,38]. Based on Kauffman *et al*. [25] criteria, out of 39 cases analysed for maize specific IgE, nine (23%) patients reflected greater than 30% binding to the GM and non GM maize antigens and were considered having significantly high maize specific IgE. Further in the analysis, using sera of five cases with clinical sensitization to maize, seven protein fractions in the range 88–28 kD were recognized as IgE binding in non GM maize antigen. Simultaneously, these 7 fractions in GM maize antigens with Cry 1Ab, Cry 1Ac and Cry 1C were also recognized as IgE binding. Thus, an overall similarity in IgE binding pattern to non GM and GM maize antigens was observed. This finding are in accordance with reported guidelines by Taylor suggesting for low levels of Cry proteins expression in transgenic foods, with allergic sensitization being more likely to occur to major or constituent proteins that exists in foods [39].

Pastorello *et al*. [40] have reported two major maize allergens as- an alpha-amylase inhibitor and a 9-kDa LTP, lipid transfer protein showing 36% and 86% binding in Italian population respectively. Other relevant allergens such as 30-kDa endochitinases A and B, 19 kDa zein-β precursor, and 26 kDa zein-α precursor have also been reported. High frequency of maize allergy in Italy has been due to high exposure to airborne maize dust, due to its high production of about 10.5 million tons per year (ISTAT official data 2008, www.istat.it).

Fasloli *et al*. [41] have identify broadly 10 protein fractions in maize extract as vicilin like embryo storage proteins, globulin-2 precursor, 50 kDa gamma-zein, endo-chitinase, thioredoxin and trypsin inhibitor. With respect to present observations, of the detected 7 IgE binding proteins on Immunoblot, it is suspected that 4 low molecular weight proteins of 28–48 kD could possibly be classified as zeins and remainder three higher molecular weight could be seed storage proteins as vicilin [40–41].

A correlation between the allergenicity of a protein and its digestibility in SGF has been suggested [21]. Out of the seven IgE binding protein fractions obtained by us, four fractions of approx. 28, 33, 41 & 62 kD have also been found to be stable proteins in the GM and non GM maize extracts as observed in the simulated gastic fluid digestion even after 1 hour.

**In conclusion**, based on *in silico* tools, it is reconfirmed that Cry 1Ab, Cry 1Ac and Cry 1C protein sequences are non allergenic sequences with no cross reactivity to known allergens and incorporation with these transgene protein sequences presents no appreciable changes in endogenous protein expression of GM and non GM maize seeds as analysed by specific IgE and Immunoblot using native maize allergic patients sera.

## Supporting Information

S1 File Table A. Food Sensitization details based on SPT and specific IgE positivity of the enrolled 39 patients. Legends—* Only three patients showed specific IgE positivity to given antigens. **Table B**. Data for specific IgE values of 39 patients and 11 healthy controls against GM and non GM maize antigens.
(DOCX)
