# Supplementary material for: Lack of Detectable Allergenicity in Genetically Modified Maize Containing “Cry” Proteins as Compared to Native Maize Based on In Silico & In Vitro Analysis
Source: PLoS One. 2015 Feb 23;10(2):e0117340. doi: 10.1371/journal.pone.0117340 (PMC4338076; doi:10.1371/journal.pone.0117340)
Supplement: S1 File — Food Sensitization details based on SPT and specific IgE positivity of the enrolled 39 patients. Legends—* Only three patients showed specific IgE positivity to given antigens. Table B. Data for specific IgE values of 39 patients and 11 healthy controls against GM and non GM maize antigens. (DOCX) [file pone.0117340.s001.docx]

**Supplementary Table A:**

| **Case No.** | **SPT positivity  ( > 2+)to different allergens** | **Specific IgE positivity to any of the commonly consumed food antigens** | |
| --- | --- | --- | --- |
|  |  | **Peanut, Soybean, Chickpea, Maize and Rice** | **Specific IgE positivity to other food antigens *** |
| 1 | Maize, Mango, Tomato | Peanut, Soybean, Chickpea, Maize |  |
| 2 | Maize, Rice | Maize |  |
| 3 | Maize | Maize, Rice |  |
| 4 | Maize | Maize |  |
| 5 | Maize, Rice | Maize |  |
| 6 | Wheat | Peanut |  |
| 7 | Peanut, Soybean, Rice | Rice |  |
| 8 | Wheat, Rice, Peanut | Peanut |  |
| 9 | Kidneybean | Chickpea |  |
| 10 | Rice | Rice |  |
| 11 | Soybean, Chickpea | Chickpea |  |
| 12 | Kidney bean, Tomato | Peanut, soybean |  |
| 13 | Tomato, Maize | Soybean |  |
| 14 | Wheat, Rice | Chickpea |  |
| 15 | Peanut, Soybean, Rice, Mustard | Rice |  |
| 16 | Milk | Peanut | Egg |
| 17 | Nuts, Sesame | Rice |  |
| 18 | Milk, Blackpepper | Rice |  |
| 19 | Egg yolk, Prawn | Chickpea | Egg |
| 20 | Almond, Milk, | Peanut |  |
| 21 | Milk, Soybean, Nuts | Chickpea |  |
| 22 | Sorghum | Peanut |  |
| 23 | Yeast, Milk, Nuts | Rice |  |
| 24 | Almonds, Vinegar, Peanut | Chickpea |  |
| 25 | Soybean, Rice, Arhar | Chickpea |  |
| 26 | Peanut, Fish, Milk | Soybean, Rice |  |
| 27 | Milk, Cashewnut | Soybean, Rice |  |
| 28 | Yeast, Almond | Soybean |  |
| 29 | Fish, peanut | Peanut |  |
| 30 | Blackpepper, Milk, Cashewnut | Chickpea | Milk, Wheat, Shrimp |
| 31 | Rice, Maize | Chickpea |  |
| 32 | Rice, Maize | Peanut |  |
| 33 | Rice, Maize | Soybean |  |
| 34 | Rice, Maize | Chickpea |  |
| 35 | Rice, Maize | Soybean |  |
| 36 | Tomato | Peanut |  |
| 37 | Tomato, Soybean | Soybean |  |
| 38 | Peanut, Blackgram | soybean |  |
| 39 | Peanut, Maize | Chickpea |  |

**Supplementary Table B:**

|  | **Non GM maize** | | **GM maize with Cry 1Ab** | | **GM maize with Cry 1C** | | **GM maize with Cry 1Ac** | |
| --- | --- | --- | --- | --- | --- | --- | --- | --- |
|  | **Patients** | **Control** | **Patients** | **Control** | **Patients** | **Control** | **Patients** | **Control** |
| 1 | 0.213 | 0.004 | 0.233 | 0.004 | 0.22 | 0.008 | 0.21 | 0.01 |
| 2 | 0.4 | 0.04 | 0.25 | 0.007 | 0.4 | 0.08 | 0.23 | 0.01 |
| 3 | 0.42 | 0.06 | 0.16 | 0.02 | 0.36 | 0.01 | 0.2 | 0.01 |
| 4 | 0.21 | 0.05 | 0.19 | 0.02 | 0.26 | 0.01 | 0.23 | 0.02 |
| 5 | 0.39 | 0.06 | 0.38 | 0.03 | 0.32 | 0.01 | 0.44 | 0.06 |
| 6 | 0.38 | 0.08 | 0.22 | 0.02 | 0.35 | 0.16 | 0.24 | 0.01 |
| 7 | 0.37 | 0.02 | 0.36 | 0.03 | 0.29 | 0.04 | 0.25 | 0.03 |
| 8 | 0.36 | 0.05 | 0.16 | 0 | 0.46 | 0.01 | 0.12 | 0.05 |
| 9 | 0.836 | 0.01 | 0.627 | 0.1 | 0.627 | 0.02 | 0.471 | 0.001 |
| 10 | 0.15 | 0.02 | 0.1 | 0.02 | 0.18 | 0.012 | 0.17 | 0.09 |
| 11 | 0.142 | 0.01 | 0.13 | 0.05 | 0.14 | 0.01 | 0.112 | 0.005 |
| 12 | 0.1 |  | 0.2 |  | 0.2 |  | 0.2 |  |
| 13 | 0.311 |  | 0.3 |  | 0.31 |  | 0.32 |  |
| 14 | 0.2 |  | 0.19 |  | 0.3 |  | 0.2 |  |
| 15 | 0.1 |  | 0.1 |  | 0.1 |  | 0.19 |  |
| 16 | 0.274 |  | 0.5 |  | 0.49 |  | 0.64 |  |
| 17 | 0.21 |  | 0.13 |  | 0.21 |  | 0.122 |  |
| 18 | 0.29 |  | 0.28 |  | 0.22 |  | 0.35 |  |
| 19 | 0.1 |  | 0.12 |  | 0.15 |  | 0.17 |  |
| 20 | 0.2 |  | 0.2 |  | 0.19 |  | 0.125 |  |
| 21 | 0.17 |  | 0.29 |  | 0.136 |  | 0.1 |  |
| 22 | 0.133 |  | 0.11 |  | 0.12 |  | 0.15 |  |
| 23 | 0.45 |  | 0.17 |  | 0.5 |  | 0.48 |  |
| 24 | 0.39 |  | 0.14 |  | 0.32 |  | 0.19 |  |
| 25 | 0.27 |  | 0.1 |  | 0.135 |  | 0.12 |  |
| 26 | 0.285 |  | 0.44 |  | 0.1 |  | 0.13 |  |
| 27 | 0.2 |  | 0.3 |  | 0.3 |  | 0.128 |  |
| 28 | 0.247 |  | 0.52 |  | 0.54 |  | 0.5 |  |
| 29 | 0.325 |  | 0.6 |  | 0.5 |  | 0.64 |  |
| 30 | 0.219 |  | 0.3 |  | 0.3 |  | 0.25 |  |
| 31 | 0.03 |  | 0.041 |  | 0.031 |  | 0.11 |  |
| 32 | 0.03 |  | 0.059 |  | 0.072 |  | 0.115 |  |
| 33 | 0.29 |  | 0.31 |  | 0.33 |  | 0.24 |  |
| 34 | 0.36 |  | 0.39 |  | 0.57 |  | 0.3 |  |
| 35 | 0.18 |  | 0.13 |  | 0.14 |  | 0.2 |  |
| 36 | 0.29 |  | 0.16 |  | 0.48 |  | 0.19 |  |
| 37 | 0.235 |  | 0.32 |  | 0.35 |  | 0.54 |  |
| 38 | 0.45 |  | 0.44 |  | 0.53 |  | 0.56 |  |
| 39 | 0.44 |  | 0.31 |  | 0.5 |  | 0.51 |  |
